# A blood safety perspective on emerging arboviral infections in the United Kingdom

**DOI:** 10.1111/tme.70041

**Published:** 2025-11-05

**Authors:** Piya Rajendra, Shannah Secret, Su Brailsford, Tanya Golubchik, Peter Simmonds, Heli Harvala

**Affiliations:** ^1^ Radcliffe Department of Medicine University of Oxford Oxford UK; ^2^ Microbiology Services NHS Blood and Transplant Colindale UK; ^3^ Big Data Institute, Nuffield Department of Medicine University of Oxford Oxford UK; ^4^ Sydney Infectious Diseases Institute (Sydney ID), School of Medical Sciences, Faculty of Medicine and Health University of Sydney Sydney New South Wales Australia; ^5^ Nuffield Department of Medicine University of Oxford Oxford UK; ^6^ Institute of Biomedicine, Faculty of Medicine University of Turku and Turku University Hospital Turku Finland

**Keywords:** arboviruses, blood safety, Dengue virus, emerging infections, Tick‐borne encephalititis virus, Usutu virus, West Nile virus

## Abstract

A core focus of the blood services is to maintain the blood supply whilst simultaneously being vigilant for potential threats to blood safety. At present, West Nile virus (WNV), Usutu virus (USUV), Dengue virus (DENV) and Tick‐borne encephalitis virus (TBEV) are considered primary arboviral threats to blood safety in the UK and Northern Europe. Climate change and globalisation have enhanced the frequency of WNV and DENV cases being reported in Europe, furthering the likelihood of their spread to the UK. Furthermore, both TBEV and USUV have already been identified in reservoir hosts in England and the first human cases of TBEV infections acquired in England have been recently documented. Existing policy to protect the blood supply against emerging viral risks is based on donor deferral or nucleic acid test (NAT) screening for those recently returning from WNV endemic areas, only. Constant evaluation of the current policy is necessary to assess the feasibility of donor deferral if the case numbers within Europe continue to increase, and to determine if selective screening for these viruses is needed. Regardless of the testing and prevention strategies decided upon by the blood services, frequent review of these policies will be necessary to reflect the national and wider disease epidemiology of these arboviral infections.

## 1 INTRODUCTION

Virus infections transmitted to humans and other vertebrates by mosquitoes and ticks (arthropod‐borne viruses, “arboviruses”) are rapidly increasing in incidence in Europe. Globalisation and climate change have extended the arboviral transmission season, increased arbovirus replication rates and expanded vector abundance and geographical range.^1^ Arboviruses commonly circulate among wild animals such as migratory birds in an enzootic replication cycle with mosquitoes and ticks, with potential spillover into humans. At a time of rapid spread of new and emerging arboviruses, there is an increasing likelihood that these infections will be imported into new geographical areas and become endemic. Health services must be prepared to recognise and diagnose these infections. Furthermore, the blood safety risk associated with them has to be considered so that adequate blood donor deferral or testing can be rapidly implemented to prevent transfusion or transplant‐associated infections.^2^

This review focusses on West Nile virus (WNV), Usutu virus (USUV), Dengue virus (DENV) and Tick‐Borne Encephalitis virus (TBEV) as these represent the current primary threats to blood safety in the UK and elsewhere in Northern Europe. WNV, USUV and TBEV have been identified in the UK in either animal hosts, vectors and/or humans. Whilst DENV has not yet been found in the UK it is currently the most rapidly spreading arbovirus among travellers and is endemic in some neighbouring European nations. The same vector mosquito species, *Aedes albopictus*, not yet established in the UK, is responsible for DENV, Zika and Chikungunya virus transmission. Imported cases of these viruses are relatively low in comparison to DENV although they require careful monitoring. Therefore, other arboviruses of potential concern to blood safety have not been addressed.

WNV, USUV, DENV and TBEV are all members of the *Flaviviridae* family that infect up to 400 million people annually. These lipid‐enveloped viruses comprise a single‐stranded positive‐sense RNA that contains one open reading frame (ORF) approximately 11 000 bases in length. The ORF encodes three structural proteins and seven non‐structural proteins. The viruses WNV, USUV and TBEV are part of the Japanese Encephalitis Antigenic serocomplex whilst DENV forms its own antigen complex, with four genetically distinct serotypes, DENV 1 to 4.^3^ The transmission of WNV and USUV is facilitated by *Culex* mosquitoes, particularly *Culex pipiens* and *Culex modestus*. WNV and USUV are maintained in an enzootic cycle between wild birds as the amplifying hosts and *Culex* mosquitoes, whereas humans are incidental dead‐end hosts.^4^ In contrast, DENV is primarily transmitted by *Aedes* mosquitoes, especially peridomestic *A. albopictus* and domestic *Aedes aegypti* that vary in their geographical distribution. Unlike in WNV and USUV, DENV is a human‐adapted pathogen, with no other known amplification host, and continues to cause some of the most devastating and explosive outbreaks in regions where DENV is established. Meanwhile, TBEV is a tick‐borne flavivirus transmitted through the bite of an infected tick such as *Ixodes ricinus* commonly found in Europe. TBEV is maintained in a transmission cycle between a range of amplification hosts, such as small mammals and birds. Humans are incidental dead‐end hosts.^5^

This review discusses the clinical significance, geographical spread and current and future considerations around the threats posed by these emerging infections to blood safety in the UK and beyond. Donor selection, the approach to screening and the risk of infection via organ transplant are considered different from the risk of blood transfusion‐transmitted infections. Therefore, organ transplantation will not be reviewed.

## 2 WEST NILE VIRUS CONTINUES TO SPREAD IN EUROPE

WNV was discovered in the West Nile district of Uganda in 1937 during a yellow fever virus surveillance program.^6^ It was, and continues to be, an underreported infection in Africa with only a few documented outbreaks reported since its identification. In the 1950s, serosurveys detected high levels of WNV antibodies in multiple African regions suggesting it was already widespread on the continent^7^, ^8^ and the first WNV outbreaks were reported in Israel and Egypt.^7^, ^9^ Almost 50 years later in 1999, WNV was first observed in the United States. It subsequently spread rapidly through the country with 4156 WNV cases reported by 47 states in 2002 and 9852 cases reported in 2003.^10^ Since 2003, the number of WNV cases has remained stable with an average of 2000 cases annually; the last major outbreak was experienced in 2012 where 5674 cases were reported.^11^

The circulation of WNV in Europe was first identified through serosurveys in Albania in 1958 and in France in 1962. The first WNV outbreak was reported from Romania in 1996 where over a three‐month period, 393 human cases of meningoencephalitis were linked to WNV.^12^ Since then, WNV cases have been increasing annually with 742 cases reported in 2023.^13^ WNV has continued to circulate in southern and central Europe but has subsequently also started to spread further north. In 2018, WNV was detected in birds and horses in Germany,^14^ followed by five locally acquired human cases in 2019.^15^ In 2020, the first locally acquired human case of WNV was identified in the Netherlands.^16^ In 2024, 1436 locally acquired human WNV infections were reported from 19 European countries demonstrating the most geographically widespread circulation of WNV in the region so far (Table 1).^17^

**TABLE 1:** Reported TBE, WNV, and DENV cases in Europe, 2023.

Country	TBE cases, 2023		WNV cases, 2023		DENV cases, 2023	
	Locally acquired	All cases	Locally acquired	All cases	Locally acquired	All cases
Austria	104	109	0	1	0	171
Bosnia and Herzegovina	NR	NR	0	0	NR	NR
Belgium	0	2	NR	0	0	218
Bulgaria	0	1	NR	0	0	2
Cyprus	NR	NR	6	6	NR	NR
Czechia	501	513	0	0	0	78
Germany	383	474	7	17	0	953
Denmark	12	28	0	0	0	102
Estonia	208	208	0	0	0	8
Greece	0	1	162	162	0	2
Spain	0	0	21	22	3	615
Finland	NR	194	0	3	0	53
France	25	36	43	48	44	2491
Croatia	0	8	0	0	0	5
Hungary	23	24	30	33	0	18
Ireland	0	0	0	1	0	20
Iceland	0	0	0	0	0	0
Italy	46	49	341	344	82	376
Liechtenstein	0	0	0	0	0	2
Lithuania	593	597	0	0	0	9
Luxembourg	0	1	0	0	0	10
Latvia	0	0	0	0	0	10
Montenegro	NR	0	2	0	NR	NR
North Macedonia	NR	0	0	3	NR	NR
Malta	0	0	0	0	0	0
Netherlands	4	7	0	0	0	75
Norway	93	112	0	0	0	61
Poland	521	523	0	0	0	70
Portugal	0	0	104	0	0	46
Romania	1	1	92	105	0	13
Serbia	NR	NR	0	92	NR	NR
Sweden	584	596	0	0	0	150
Slovenia	63	63	NR	0	0	9
Slovakia	142	143	NR	0	0	6
Kosovo	NR	NR	NR	0	NR	NR
UK	0	NR	0	NR	0	576
Total EU/EEA (without UK)	3303	3690	714	742	129	5573

*Note*: NR‐ not reported during this time period. Data accumulated from the European Centre for Disease Prevention and Control's Surveillance Atlas of Infectious Diseases.

A total of 10 confirmed travel‐acquired WNV human cases have been reported since 2019 in the UK. These cases include three that travelled to the USA, two to Canada, one each to Egypt, Hungary, South Africa, Eastern Europe and one individual to more than one destination. Of the infected persons three case presentations were reported: a 59 year old with neuroinvasive WNV after travel to the US and a couple in their 70s with meningoencephalitis and influenza‐like symptoms respectively, after travel to South Africa.^18^, ^19^ However, no autochthonous (locally acquired) human WNV cases have been reported in the UK to date. WNV was recently detected in *Aedes vexans* mosquito pools collected in Nottinghamshire, England in July 2023.^20^ The virus has not been detected in other mosquito species, horses or birds in the UK.^21^ However, in 2010, it was discovered that *C. modestus* mosquitoes had begun establishing populations in the south‐east of England.^18^ This is concerning, as this species can be infected with low levels of virus and has been known to transmit the virus.^22^ Furthermore, migratory birds have been implicated in the spread of WNV to new locations.^23^ Regions where *C. modestus* has recently spread overlap with migratory bird routes from Africa.^24^

### 2.1 Clinical significance

Approximately 80% of WNV cases are asymptomatic with the remaining 20% developing fever and 1% severe neurological disease. The elderly and immunocompromised individuals are considered at high risk of severe infection.^25^ WNV infections have been a notifiable disease in the EU since 2018; the member states are required to report the number of confirmed WNV cases to the ECDC.^26^ Transfusion transmission of WNV was first reported in 2002, when active blood donor surveillance was established in the United States.^25^ That year saw 4156 clinical WNV infections^27^ and 23 transmissions of WNV infections via blood transfusion with one recipient developing a fatal neuroinvasive disease.^28^ As a result, blood donor screening for WNV RNA in pools up to 24 was urgently implemented. Although this mini‐pool strategy intercepted over 1000 potentially viraemic blood donations during 2003–2004^29^ it failed to entirely prevent transmission via blood transfusion, with infections occurring in several recipients of blood units with low viral loads that were missed by the pooled screening.^30^ While pooled screening is often done in non‐endemic regions, or in endemic regions before the first human, animal or vector cases of the season are identified, more sensitive individual donation screening is required to effectively prevent WNV transmission.^27^, ^31^ WNV transmission via blood transfusion has also been reported in two European countries, Italy and Greece, where WNV is known to be endemic.^32^

## 3 USUTU VIRUS—ALWAYS ASYMPTOMATIC INFECTION OR CAUSE FOR CONCERN?

USUV was identified in mosquitoes in South Africa in 1959 and has since been detected in several other African countries. The first human cases were reported in the Central African Republic in 1981, and Burkina Faso in 2004. Both of these cases were febrile infections without neurological symptoms.^33^ The virus was first reported in Europe after a USUV outbreak in Austrian blackbirds in 2001, but retrospective studies have indicated the virus had been in circulation in Italy since 1996. Since its first discovery in Europe, it has been found in avian hosts and mosquito vectors in 15 European countries.^34^

Human USUV infections have been reported in Italy, Germany, Croatia, Austria, Hungary, Czech Republic, Netherlands, France and Switzerland (Table 2).^34^ The first human USUV infection in Europe was identified in 2009.^35^ Most USUV infections have been asymptomatic and found among healthy blood donors in Italy, Hungary and Germany.^36^ However, USUV can, in some cases, cause a severe neuroinvasive infection particularly in the immunocompromised and the elderly host.

**TABLE 2:** USUV infections detected in blood donors and reported clinical neurological cases from 2008 to 2023.

Country	PCR positive cases (blood donors)	Serology positive cases (blood donors)	Clinical neurological cases
Italy	38	56	13 neurological cases including one fatality
Croatia	None reported	None reported	6 neurological cases including one fatality
France	None reported	None reported	2 neurological case
Czech Republic	None reported	None reported	1 neurological case
Hungary	None reported	5	1 neurological case
Austria	24	None reported	1 neurological case
Switzerland	None reported	None reported	1 neurological case
Netherlands	7	7	None reported
Germany	1	1	None reported

**FIGURE 1:**
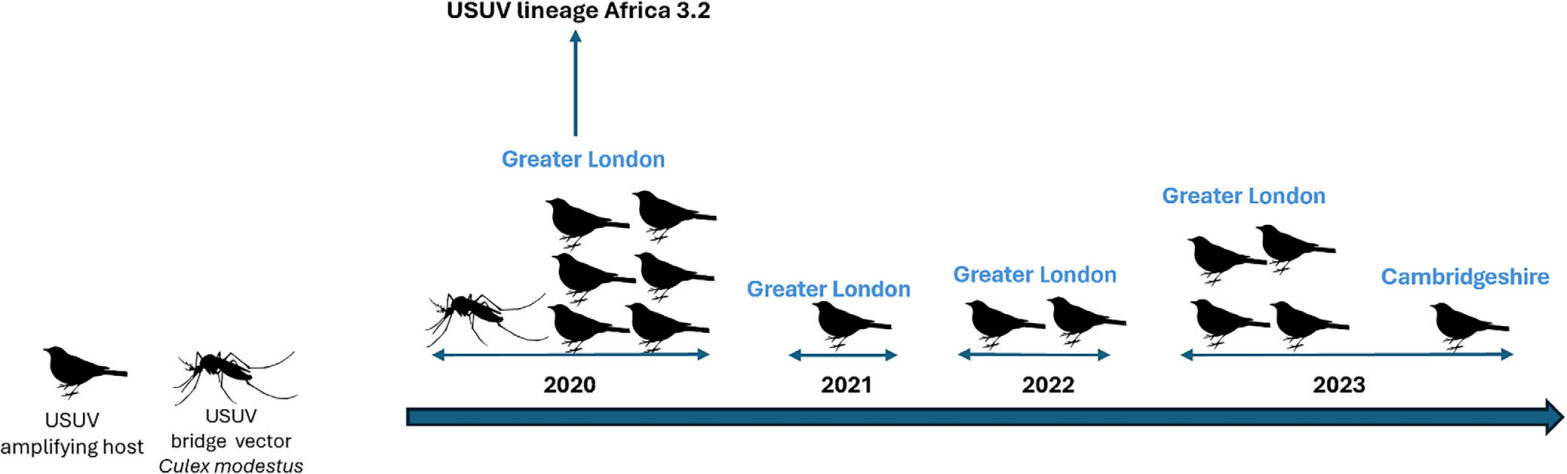
Timeline of detection of USUV in bird and mosquitos in England, 2020–2023.

Recent extensive surveillance of mosquitos and birds has identified the presence of USUV in vector populations in England (Figure 1). It was first identified in the UK in the summer of 2020, in black birds in the Greater London area. Since then, USUV‐positive birds have also been identified in the same region but also in Cambridgeshire.^37^

Sequence analysis of USUV in non‐migratory birds and mosquitoes revealed near identical genome sequences being found each year, suggesting probable overwintering of the virus in England.^38^ To date, no human cases of USUV have been identified in England.

### 3.1 Clinical significance

To date, a total of 31 symptomatic (neurological conditions and febrile illness) USUV infections, of which two were fatal, have been reported in Europe.^39^ Most human USUV infections have been reported by Italy; it is the only country where human USUV infections are notifiable. A joint surveillance programme for USUV and WNV was established in Italy in 2017. Based on their surveillance, around 10% of USUV infections were symptomatic (3 from 33).^40^ Although transmission of USUV by blood transfusion has not been reported to date, the findings of high transient viraemia after infection and a high proportion of asymptomatic infections suggest this is likely.

## 4 TICK‐BORNE ENCEPHALITIS VIRUS—IS THERE A RISK?

TBEV is endemic in most European countries, with a total of 28 680 confirmed TBEV cases reported by 29 countries during 2013–2022.^41^ It has also recently spread to new countries, the Netherlands and the UK.^42^, ^43^ Furthermore, cases appear to be on the rise with increased numbers recently reported in Austria, Czechia, Finland, France, Germany, Norway, and Poland.^44^

**FIGURE 2:**
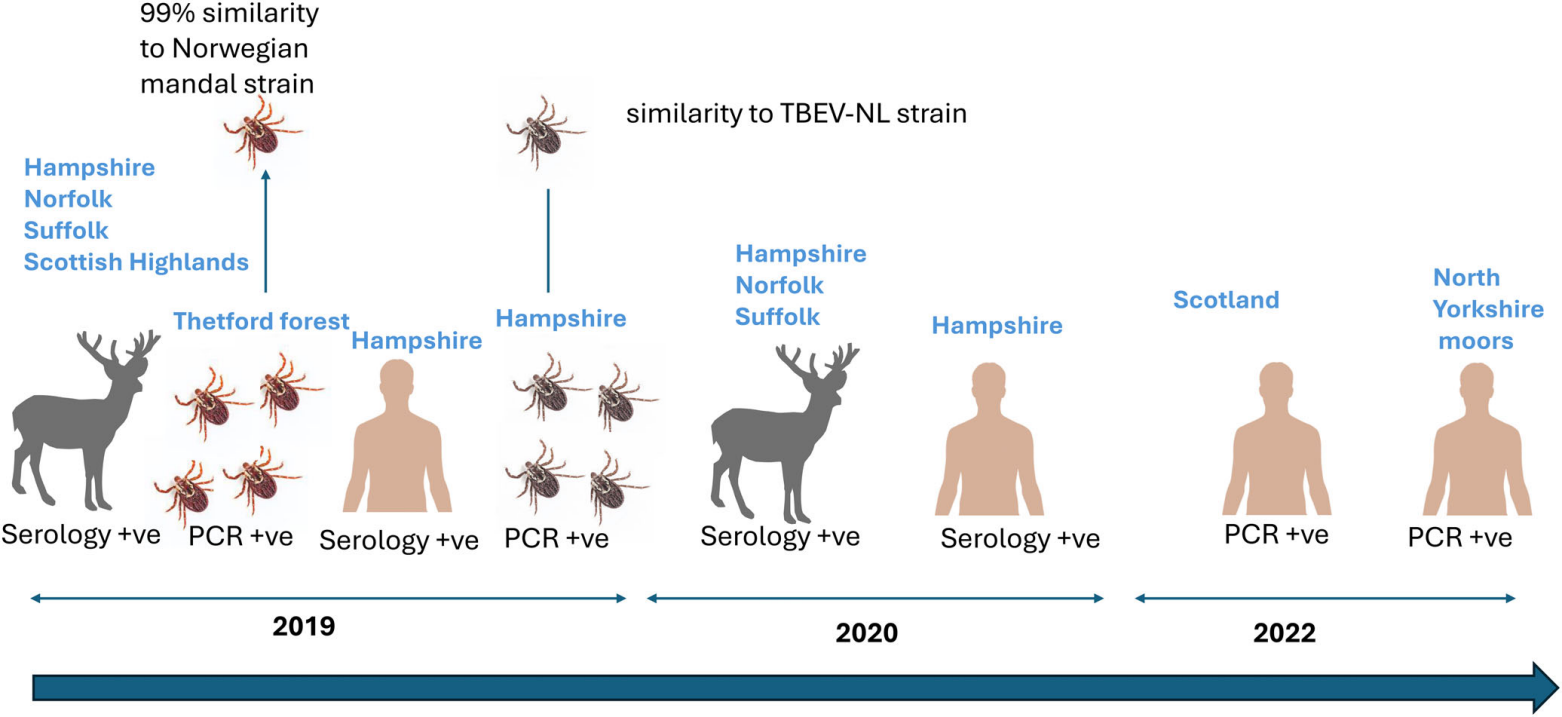
The detection of TBEV antibodies and RNA detection in roe deer, ticks and humans in the UK from 2019 to 2022, based on published data.^42^ Deer image indicates serological surveillance for TBEV was conducted. Tick images denote tick pools were tested for TBEV. A human figure indicates an identified human case. Locations of where TBEV was found is highlighted by the blue text above each image.

Although the *I. ricinus* vector has been known to be widespread in the UK for decades, the first locally acquired human TBEV case was reported in Hampshire in 2019.^45^ This first human UK case developed meningoencephalitis. There were three subsequent cases reported in Scotland and North Yorkshire in 2022 for which no clinical details are available (Figure 2). In parallel, seven travel‐related TBEV cases were reported in the UK between 2014 and 2018.^46^

The first UK human TBEV case prompted seroprevalence studies in deer, the known reservoir host for the virus in England and Wales, 2018–2019.^35^ A total of 1309 culled deer were included in the study, and 52 deer were found seropositive for TBEV. The seroprevalence was highest in Norfolk (51.4%), followed by Hampshire (14.3%), Suffolk (10.7%), and Scotland (8.6%). Five from 2041 individual *I. ricinus* ticks plucked from culled deer from the Thetford Forest region in Norfolk tested positive for TBEV RNA by PCR (0.2%). These investigations demonstrated TBEV is in circulation in several deer populations in various geographical locations in the UK. Furthermore, the full‐length genome sequence isolated from ticks in Thetford Forest in 2019 was most closely related to the Norwegian Mandal strain from 2009 whilst the strain identified from ticks in Hampshire in 2019 was closely homologous to the TBEV‐NL‐Salland strain first identified in the Netherlands, 2017.^47^ This indicates that more than two different TBEV strains have been introduced and are circulating in the UK. Furthermore, migratory birds have likely contributed to the introduction of TBEV strains in the UK.

The epidemiology of TBEV is influenced by vaccination that is variably used in different European countries. For example, Austria has implemented a universal TBEV vaccination with a very high vaccination rate whilst vaccinations in Sweden are targeted to people living in TBEV‐endemic regions only.^48^ In 2022, these strategies resulted in a greater than two fold difference in confirmed TBE cases [206 per 100 000 population in Austria versus 465 per 100 000 population in Sweden^49^].

### 4.1 Clinical significance

Of the known subtypes, the European subtype (TBEV‐EU) is most common in Europe. It is associated with a milder disease, with symptomatic infection in 20%–30% and a case fatality rate of 1%–2%. Most TBEV infections are biphasic. The first viraemic phase lasts for around 5 days and is associated with non‐specific symptoms such as mild fever, fatigue, headache and nausea. The second phase may follow approximately a week later, with the involvement of the central nervous system.^5^ Usually, TBEV infections are only clinically recognised and diagnosed if they reach this second phase. Although transmission of TBEV via blood transfusion is considered rare, it is plausible as individuals are known to be viraemic during the first phase of infection.^44^ The only reported transmission was from a blood donor who had visited Kumlinge Island, Finland.^50^ During this time, TBEV seroprevalence among blood donors in the region of Kumlinge was 5%.^51^ Recipients of the blood donation were followed up and one recipient experienced a febrile illness while the other recipient developed neurological symptoms. Their TBEV diagnosis was made by serology, limiting the possibility of confirmatory testing and sequence comparisons. Although TBEV has been a notifiable disease in Europe since 2012, the true incidence is likely higher than reported due to infections being clinically inapparent or associated with non‐specific symptoms and a lack of routine diagnostic screening of possible cases and/or contacts.^52^

## 5 DENGUE VIRUS—POTENTIAL TO SPREAD RAPIDLY

In 2024, over 14 million DENV cases with 10 000 deaths were reported from 120 countries, most of them from the Americas (90%).^53^ Both imported and locally acquired DENV cases are increasing in Europe, with autochthonous transmission occurring in France (83 cases), Italy (213 cases) and Spain (8 cases) in 2024.^54^ This is likely exacerbated by the spread of invasive mosquito species, *A. albopictus*, which has adapted to overwinter in more temperate regions. This has led to its establishment in 13 European nations in 2023 compared to 8 EU/EEA nations in 2013.^55^ Although *A. albopictus* is not yet permanently established in the UK, multiple introductions have occurred in southeast England between 2016 and 2019, and 2023.^56^ To date, DENV has not been identified in mosquitoes in the UK. However, it has been demonstrated that once *A. albopictus* is established in a region, local outbreaks of arboviruses such as DENV commonly follow 5–15 years later.^57^ Climate modeling has proposed that London, and its surrounding areas, have a suitable climate for the survival of *A. albopictus*, suggesting it could become established here in the next 15 years.^58^

In the UK, all DENV cases have been travel‐related only. Between January and June 2024, there were 473 dengue cases identified in returning travellers across England, Wales, and Northern Ireland. Most of the reported cases travelled to Barbados (28%), followed by Brazil (15%) and Indonesia (12%).^59^

### 5.1 Clinical significance

Most DENV infections are not easily clinically diagnosed, as the acute infections usually display non‐specific symptoms such as high fever, headache, body aches, nausea and rash only. Around 20% of cases will develop more severe disease, sometimes leading to death. Unique to DENV is its antibody‐dependent enhancement of infection, where more severe infection may occur in those who have a secondary infection with a different dengue type.^60^

DENV transmission through blood transfusion has been well documented; reports originate mainly from endemic countries such as Hong Kong, Singapore, Puerto Rico and Brazil.^61^, ^62^ To date, there have been 8 transfusion‐transmitted DENV cases that have caused symptomatic infections in recipients. All affected individuals suffered comorbidities; however no fatalities were noted.

## 6 CURRENT AND FUTURE MEASURES TO PREVENT ARBOVIRUS TRANSMISSION BY TRANSFUSION

### 6.1 Donor deferral and selective screening

While human arbovirus infections are rare in the UK and locally acquired infections even rarer, current measures to prevent transmission revolve around donor deferral or donation screening for those recently returning from endemic areas. These measures are determined by the Joint UKBTS Professional Advisory Committee (JPAC) policies and the geographical disease risk index. For this, a joint epidemiology unit between NHSBT and the UK Health Security Agency (UKHSA) produces a monthly emerging infections report (EIR). The information in the EIR is analysed by the Standing Advisory Committee on Transfusion Transmitted Infection (SACTTI) and potential risks to the safety of donated blood identified are graded based on the urgency of action required. Horizon scanning also reports on epidemiological changes of known infectious agents that may alter the geographical disease risk for the deferral of blood donors.

In England, returning travellers are screened for WNV RNA in mini‐pools of 6. Between 2012 and 2023, 3 WNV RNA positives were identified by screening of 415 000 donations from donors who disclosed recent travel to an endemic area.^63^ As USUV tends to co‐circulate in the same geographical regions as WNV, the potential risk of USUV transmission is largely mitigated by similar deferral or by WNV RNA screening which also detects the genetically closely related USUV.^64^

For TBEV, there is no blanket deferral or testing policy for returning travellers. However, blood donors are enquired whether they have experienced a recent tick bite but noting this is also incomplete and likely a non‐effective way to screen those exposed. This policy is informed by observations of extremely infrequent transmission and relatively low seroprevalence of TBEV in endemic areas such as Austria and Scandinavia.^27^, ^65^ The current surveillance in the UK indicates that TBEV is already widely distributed in the *I. ricinus* vector and in deer reservoir hosts.^43^

**FIGURE 3:**
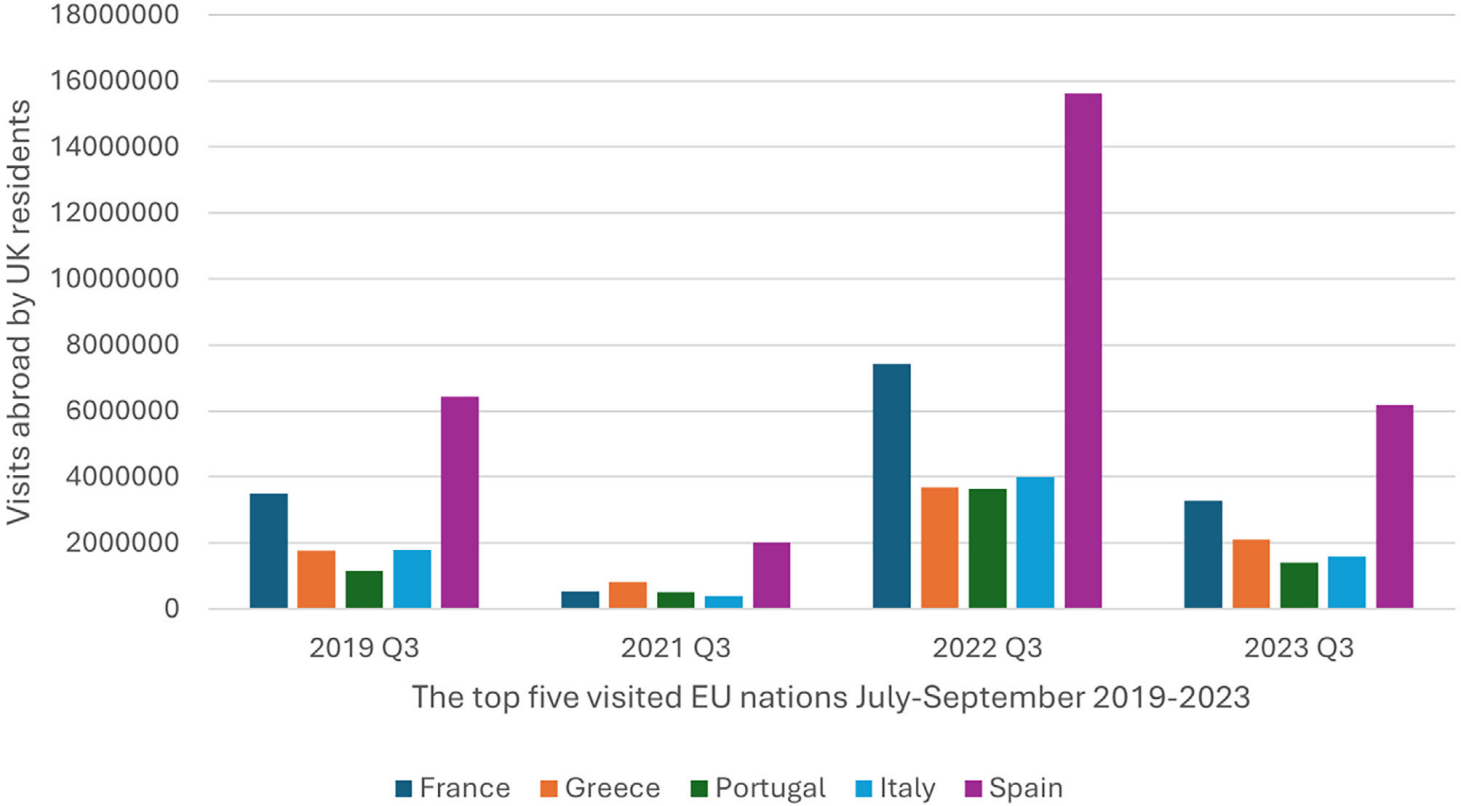
The most visited European nations by UK residents in July–September (Q3), 2019–2023. The year 2020 is omitted as data was not submitted during the COVID‐19 pandemic (Data from the national statistics office UK).

For DENV, donors who report recent travel to DENV‐endemic areas are excluded from blood donation for 6 months from their return to the UK if they had symptoms consistent with Dengue or they have been diagnosed with DENV, or for 4 weeks from their return if they have had no symptoms. This policy may have a significant impact on the UK blood supply if DENV continues to spread within these countries and beyond; Spain, France and Italy, being visited over 10 million times by UK residents in 2023, are also the three countries with the most extensive circulation of DENV in Europe (Figure 3).^66^ Similar to WNV, regular monitoring of European and worldwide surveillance data is needed to anticipate and respond to the rapidly changing epidemiology of DENV to ensure an effective deferral of donors returning from at‐risk areas. This is particularly relevant not only for risk reduction but also to balance against potential increasing blood donor loss in travellers to areas of changing DENV incidence.

Current deferral and selective testing measures must be actively reviewed in response to the potential establishment of WNV, USUV, TBEV and DENV circulation in the UK or elsewhere in Europe. The UK Health Security Agency (UKHSA) collects entomological data on vector species distributions and their virus carriage, veterinary screening of bird and animal populations and conducts rigorous investigation of infection sources in diagnosed cases of human arbovirus infections. This provides essential monitoring data on the spread of arboviruses into the UK and their associated vectors. This will be supplemented in the future by geographically targeted serological screening of donors in the NHSBT CODONET programme (IRAS ID 335458).

## 7 FUTURE MEASURES

The identification of significant autochthonous transmission within the UK of any of the vector‐borne viruses considered in the review would necessitate the introduction of targeted screening or other means to remove the infectivity of blood components.

Viral and bacterial inactivation methods using methylene blue and visible light could be used by the blood services to prevent arbovirus transmission. Pathogen reduction has proven effective for arboviruses such as DENV and WNV^67^, ^68^ and has obtained regulatory approval for use for treatment of platelets but not for red cell components. However, in 2022, the UK Advisory Committee on the Safety of Blood, Tissues and Organs (SaBTO) advised against their usage in the UK on the grounds of cost, loss of the quality of platelets and the current effectiveness of bacterial screening that outweighed its benefits of increased shelf‐life and ability to inactivate all viruses currently unscreened by NAT or serology.^69^ This advice will no doubt be reviewed in the future, particularly if licensed for use on red cells and with better demonstrated cost‐effectiveness.

An alternative option would be the introduction of universal donor testing for arbovirus viraemia using multiplexed NAT,^70^ as an additional test within the current HIV‐1, HBV, HCV and HEV NAT screening framework. Its effectiveness will be crucially dependent on assay sensitivity—while current screening in pools of 24 would identify and exclude most arbovirus‐viraemic donations, past experience with WNV screening in the US has demonstrated that transmission can occur even from units negative on screening with a pool size of 6,^28^ and this may similarly limit the effectiveness of screening for other arboviruses.

In the longer term, technology developments in next generation sequencing and blanket genetic characterisation of the donor “virome” may scale up and possess sufficient sensitivity and specificity to enable blood donor screening for emerging pathogens including arboviruses. The use of targeted enrichment such as by multiplex PCR or probe‐based capture prior to sequencing could remove the barrier of reduced sensitivity of untargeted metagenomics.^71^, ^72^ Metagenomic screening has been trialled in a single centre in the UK for the diagnosis of viral infections in febrile returning travellers,^73^ although further optimisation would be required in areas of assay sensitivity and reduction in cost to be a practical option for screening on the scale required by the blood services.

## 8 CONCLUSIONS

Over the next century, climate forecasters predict that approximately 1 billion people, predominantly in Europe and subtropical regions, will be infected by a mosquito‐borne virus for the first time.^74^ The viruses discussed in this review are becoming increasingly common in Europe, and the blood services must be prepared for their likely spread and emergence. While WNV and DENV continue to spread in Europe they are yet to reach the UK; USUV and TBEV are already known to circulate in England and are likely more widely spread than currently recognised.

The blood services in the UK and elsewhere in Europe have the unenviable task of maximizing blood safety, in this case through protecting recipients from arbovirus infections while at the same time maintaining the blood supply and controlling costs of screening and component processing; measures taken must lie within acceptable boundaries for cost‐effectiveness. These factors crucially depend on future incidences of arbovirus infections in the UK and elsewhere, the extent to which risk factors for infection can be identified to enable deferral or selective testing, and the effectiveness and costs of NAT screening and pathogen reduction. At a time of rapid change in climate and distributions of viruses and vectors, as well as changes in societal expectations of blood safety, these are all very much moving goalposts. Testing and prevention strategies adopted by the blood services will require ongoing and proactive review to ensure the longer‐term safety of transfusion.

## AUTHOR CONTRIBUTIONS

Conceptualisation: Heli Harvala. Funding acquisition: Peter Simmonds, Heli Harvala. Writing – original draft: Piya Rajendra. Writing – review and editing: all authors. Supervision: Heli Harvala. All authors approved the final version.

## FUNDING INFORMATION

This work was supported by the National Institutes for Health and Care Research [grant number NIHR203338]. The funding body had no role in the study's design, data collection, analysis, or manuscript writing.

## CONFLICT OF INTEREST STATEMENT

The authors have no competing interest.

## PATIENT CONSENT STATEMENT

Patient data was not used in this review; therefore consent was not required.

## Data Availability

Data sharing not applicable to this article as no datasets were generated or analyzed during the current study.
